# Early- and Late-Luteal-Phase Estrogen and Progesterone Levels of Women with Premenstrual Dysphoric Disorder

**DOI:** 10.3390/ijerph16224352

**Published:** 2019-11-07

**Authors:** Ju-Yu Yen, Huang-Chi Lin, Pai-Cheng Lin, Tai-Ling Liu, Cheng-Yu Long, Chih-Hung Ko

**Affiliations:** 1 Department of Psychiatry, Kaohsiung Municipal Ta-Tung Hospital, Kaohsiung Medical University, Kaohsiung 801, Taiwan; yenjuyu@cc.kmu.edu.tw (J.-Y.Y.); superfoxcat@yahoo.com.tw (P.-C.L.); 2 Department of Psychiatry, Faculty of Medicine, College of Medicine, Kaohsiung Medical University, Kaohsiung 807, Taiwan; cochigi@yahoo.com.tw; 3 Department of Psychiatry, Kaohsiung Medical University Hospital, Kaohsiung 807, Taiwan; dai32155@gmail.com; 4 Department of Obstetrics and Gynecology, Kaohsiung Municipal Siaogang Hospital, Kaohsiung Medical University, Kaohsiung 812, Taiwan; urolong@gmail.com; 5 Department of Psychiatry, Kaohsiung Municipal Siaogang Hospital, Kaohsiung Medical University, Kaohsiung 812, Taiwan; 6 Research Center for Environmental Medicine, Kaohsiung Medical University, Kaohsiung 807, Taiwan

**Keywords:** PMDD, women, estrogen, progesterone, luteal, prospective

## Abstract

Objective/introduction: The dynamics of ovarian hormone fluctuations during the luteal phase of the menstruation cycle were previously suggested to contribute to the development of premenstrual dysphoric disorder (PMDD) symptoms, but adequate empirical evidence has not been obtained from hormone concentration studies. We prospectively evaluated estrogen and progesterone levels in the early luteal (EL) and late luteal (LL) phases in women with PMDD and the association of these levels with PMDD symptom severity. Methods: 63 women with PMDD and 53 controls without such severe symptoms were evaluated for the estrogen and progesterone levels, and PMDD severity in the EL and LL phases. Results: The results demonstrated that the women with PMDD had a lower EL-phase estrogen level than the controls. Covariant analysis demonstrated that the interaction term between EL-phase estrogen and EL-phase progesterone level was associated with PMDD severity. Among women with lower EL estrogen levels, higher EL-phase progesterone was observed among the women with PMDD versus controls. These results suggest that low EL-phase estrogen level could moderate the provoking effect of EL progesterone in women with PMDD. Overall, these data suggest a possible role of estrogen and progesterone in the development of PMDD symptoms.

## 1. Introduction

Premenstrual dysphoric disorder (PMDD) is listed as a depressive disorder in the Diagnostic and Statistical Manual of Mental Disorders, Fifth Edition (DSM-5). A previous review suggested that the prevalence of PMDD ranges from 1.2% to 6.4% [1]. Women with PMDD experience predictable and cyclic psychological, behavioral, and somatic symptoms that are aggravated approximately 6 days during the late luteal (LL) phase, are improved after the onset of menses, and reoccur throughout most of the reproductive years [2,3,4]. The LL onset of PMDD symptoms suggests that fluctuating ovarian hormones play a role in its mechanism [5].

The symptoms of PMDD disappear during spontaneous anovulatory cycles, pregnancy, and post menopause. Furthermore, women with PMDD reported improved symptoms when endogenous ovarian hormone secretion was suppressed with gonadotropin-releasing hormone (GnRH)-agonist treatment [6] or hormonal contraception [7]. These results suggest that the menstrual cycle is required for the development of PMDD symptoms. Premenstrual syndrome (PMS)-like symptoms reoccur in postmenopausal women who receive sequential hormonal replacement therapy [8]. The add-back of estrogen or progesterone induces premenstrual symptoms among women with PMDD under GnRH-agonist-induced anovulation but not among women without PMDD [6,9,10]. These reports suggest that ovulation and ovulation-related processes trigger premenstrual symptoms among women with this vulnerability [11]. Thus, ovarian hormones that fluctuate during the menstrual cycle, such as estrogen and progesterone, were suggested to play an essential role in the mechanism of PMDD [12]. However, aside from interventional studies, investigations on ovarian hormone levels during natural menstrual cycles have mostly failed to find a difference between women with PMDD and those without [13].

In general, progesterone level remains stable during most of the mid-luteal phase, before declining sharply prior to menses [14]. Progesterone withdrawal was suggested to contribute to PMDD symptoms in an animal study [15]. In line with this hypothesis, higher progesterone was associated with lower irritability and symptoms of fatigue in healthy women [16]. By contrast, add-back of progesterone provoked premenstrual symptoms among women with PMDD under ovarian suppression [6], but not in controls. The level of allopregnanolone, a metabolite from progesterone, decreased after premenstrual symptoms were reduced [17,18] and was associated with a blunted hypothalamus–pituitary–adrenal axis function in PMDD [19]. Thus, the pathological response to both withdrawal from and exposure to progesterone or allopregnanolone was suggested to contribute to PMDD symptoms [1,12].

Estrogen has been extensively investigated to determine its effect on depression in women [20,21]. The estrogen receptor alpha gene polymorphism is associated with the risk of PMDD [22] and moderated the association between estrogen and emotional regulation [23]. Thys-Jacobs et al. [24] demonstrated a low free luteal estradiol level among women with PMDD. The brain inflammation had been suggested to be a factor contributing to PMDD [25]. The anti-inflammatory effect of estrogen was suggested to play a role in the link between estrogen drop and premenstrual symptoms in the luteal phase. However, most studies have failed to support the hypothesis of a role for excesses or deficiencies of progesterone or estrogen in PMDD [26].

In the luteal phase, the corpus luteum secretes primary progesterone. After a short drop in estrogen, both estrogen and progesterone increase and reach a peak around the mid-luteal phase, after which they rapidly decline in the LL phase to contribute to menstruation [27]. Thus, women with PMDD may have altered sensitivity to ovarian hormone dynamics, such as when the estrogen and progesterone levels increase early in the luteal phase, peak during the mid-luteal phase, or decrease to basal levels during the LL phase. Thus, investigating only one time point in the luteal phase is unable to demonstrate ovarian hormone dynamics. Although PMDD symptoms peak before menses, both estrogen and progesterone are at a low level, and the difference was difficult to discover. Moreover, the association between ovarian hormone and PMDD symptoms should be evaluated separately among women with PMDD and healthy women because of the difference in vulnerability. Lastly, the delayed effect of an ovarian hormone, if it exists, could bias the result of cross-sectional studies.

Thus, we hypothesized that ovarian hormone dynamics during the luteal phase have an essential role in the development of PMDD symptoms. We aimed to evaluate estrogen and progesterone levels in the early luteal (EL) and LL phases in women with PMDD and to determine their association with LL-phase PMDD symptoms severity. This study explored the role of luteal-phase estrogen and progesterone concentration in PMDD symptoms.

## 2. Methods

Ethical code from IRB (the Institutional Review Board of Kaohsiung Medical University Hospital, Taiwan): KMUHIRB-SV(I)-20150040.

### 2.1. Participants

Participants were recruited at university campuses through advertisements looking for women with untreated PMDD and controls without such severe symptoms. Volunteers for the PMDD group were required to report at least five symptoms of PMDD criteria [2], with most symptoms being alleviated after the onset of menses. The control group volunteers were required to report one or fewer symptoms or to have two or more mild symptoms without functional impairment. Individuals currently taking psychotropic or gonadotropic medication were excluded.

After obtaining informed consent, 88 participants fulfilled the inclusion criteria in the PMDD group and 68 in the control group. The participants were interviewed by a psychiatrist to exclude psychotic disorder, illicit substance or alcohol use disorder, and bipolar I disorder using the Mini-International Neuropsychiatric Interview [28] and to diagnose PMDD using the DSM-5 criteria [2]. This resulted in 81 participants in the PMDD group and 68 participants in the control group. Of these, 63 women with PMDD and 53 healthy participants were included in the final analysis after elimination of women with irregular menstrual cycles or unfulfilled symptomatic cycles during testing, as described in a later section. This study was approved by the Institutional Review Board of Kaohsiung Medical University Hospital.

### 2.2. Measures

#### DSM-5 PMDD Diagnostic Criteria 

The diagnostic criteria were as described. First, the presence of at least five of the following symptoms in most menstrual cycles within the preceding year, among marked affective lability, irritability, depressed mood, anxiety, lack of concentration, lethargy, overeating, sleep problems, sense of being overwhelmed, and physical symptoms occurring during the final week before onset of menses; these symptoms start disappearing within a few days after onset and are minimal or absent in the week after menses. Second, the presence of at least one of the first four mood symptoms. The symptoms are associated with clinically significant distress related to work, school, social activities, or relationships, confirmed based on prospective daily ratings during at least two symptomatic cycles [2]. The prospective ratings are mentioned in the Procedures section.

PMDD symptom severity was assessed using the PMDD severity questionnaire (PMDDSQ). We developed a scale to rate the severity of symptoms experienced at the time of answering the questionnaire, with reference to the 11 PMDD criteria in the DSM IV-TR [29]. Each symptom of PMDD criteria, such as depressed (item 1) or irritable (item 4), was rated using an 11-point Likert scale (0, “completely unnoticeable” to 10, “extremely severe”), and the total scores were analyzed. The Cronbach α for the questionnaire was 0.98, and the 4 week test–retest reliability was 0.92 [30].

For assessment of estrogen and progesterone levels, blood samples were obtained directly from each participant through a cannulated vein for the assessment of various parameters during the EL and LL phases. The serum was separated out through centrifugation and then stored at −20 °C until further analysis. Estrogen and progesterone levels were measured using a Coat-A-Count Radioimmunoassay Kit (SIEMENS Medical Solutions Diagnostics, Los Angeles, CA, USA). The inter-assay variation coefficient was 8.1%, whereas the intra-assay variation coefficient was 7%.

### 2.3. Procedures

The participants were tested in both the EL phase (3 or 4 days after ovulation, as predicted using the last menstruation cycle) and the LL phase (3 days before the predicted onset of menstruation) in the same menstrual cycle. To minimize variability in circadian rhythm or oral intake, the participants arrived at the laboratory room for the blood test in the morning without having eaten (up to 300 mL water was permitted).

Daily record, such as Daily Record of Severity of Problems, could reflect the onset and the resolution of symptoms during the menstrual cycle. However, it is time consuming and difficult to check the record every day in study. Previous study had demonstrated that weekly evaluation of the Reported-Outcomes Measurement Information System through Computerized Adaptive Testing showed the same systematic changes in the menstrual cycle as the daily record did [31]. Thus, in addition to being administered during EL and LL phases in the evaluating cycle, the PMDDSQ was administered once a week for the two subsequent menstrual cycles. Symptomatic cycles were defined as: LL-phase score 30% higher than the minimal score during the balance of the menstrual cycle [32]. Data from the participants in the PMDD group that fulfilled the symptomatic cycle criterion for two or more consecutive menstrual cycles were included in the analysis.

### 2.4. Statistical Analysis

Because the estrogen, progesterone, LH, and FSH (follicle-stimulating hormone) levels were not normally distributed in our sample, the difference in EL level or LL level between the women with PMDD and controls was evaluated using the Mann–Whitney U test. Nonparametric analysis could provide robust results for testing these differences. The associations of estrogen and progesterone levels with PMDD symptom severity were tested using a univariate general linear model (GLM). Repeated-measures analyses of variance (ANOVA) was used to evaluate the severity of PMDD, with menstrual cycle phase (the eight evaluation points of the PMDDSQ in the menstrual cycle) and PMDD diagnosis (PMDD group versus control group) as influential factors. A *p*-value <0.05 was considered statistically significant for all analyses.

## 3. Results

13 controls and eight women with PMDD were excluded because their menstruation occurred ≥2 days earlier or ≥7 days later than their LL assessment day. An additional eight women with PMDD were excluded because they did not meet the criteria for two consecutive symptomatic cycles. The total analysis population excluded two women with PMDD and two controls who did not complete the blood sample collection, leaving 63 women with PMDD and 53 controls in the final analysis groups. The participants were similar in age and years of education (Table 1). There was also no difference in EL and LL evaluation phases (evaluated date/menstrual cycle duration of the evaluation cycle) between the PMDD and control groups.

### 3.1. Luteal Estrogen and Progesterone Levels in Women with PMDD

**Figure 1:**
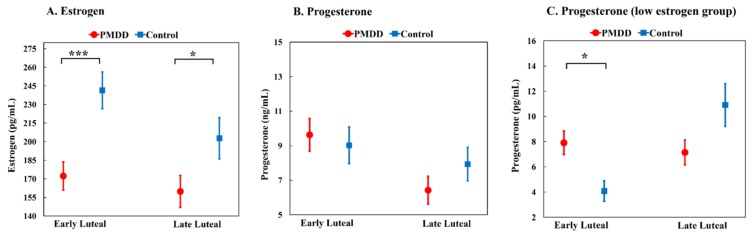
The early luteal (EL) and late luteal (LL) estrogen and progesterone levels among premenstrual dysphoric disorder (PMDD) group and control group and those among low EL estrogen participants. *: *p* < 0.05; ***: *p* < 0.001; The PMDD group had lower EL and LL estrogen level than the control group; The PMDD group had a higher EL progesterone level than the control group among participants with lower estrogen (<203.9 pg/mL).

The PMDD group had significantly lower estrogen levels versus the controls in the EL phase (*p* < 0.001; Table 2 and Figure 1A). Their LL estrogen levels were also significantly lower than those of the controls (*p* = 0.026). No significant difference in progesterone levels was discovered. There was a significant association between the estrogen and progesterone levels in the EL and LL phases in the PMDD group in Table 3. This was expected because both estrogen and progesterone are mostly secreted by the corpus luteum in the luteal phase.

### 3.2. EL Estrogen Level of Women with PMDD

Because EL-phase estrogen was the hormone most strongly associated with PMDD and was also associated with EL progesterone in this study, we next evaluated the effects of EL-phase estrogen and progesterone levels on LL-phase PMDD severity using the univariate GLM. The result demonstrated that EL estrogen was significantly associated with LL-phase PMDD severity (Model 1 in Table 4). We included interaction term of EL estrogen and progesterone in the GLM model. The EL-phase progesterone level and interaction term were significantly associated with PMDD severity. This suggests that EL-phase estrogen level interacted with EL-phase progesterone level in their association with PMDD severity. Progesterone level was also discovered to have a role in PMDD severity when controlling its interaction with estrogen level. We further tested the GLM model for the PMDD severity in the PMDD group. The results repeatedly demonstrated that EL-phase progesterone level and the interaction term were significantly associated with PMDD severity. Based on the concept of Baron and Kenny [33], the significance of the interaction term might suggest the moderating role of low EL estrogen. Furthermore, this result demonstrated an effect of EL-phase progesterone level on PMDD severity with control of the moderating effect of estrogen among women with PMDD.

To determine the possible moderating role of EL-phase estrogen level in the effect of progesterone on PMDD, we classified the participants into a high-estrogen group and low-estrogen group based on the mean EL-phase estrogen level (203.9 pg/mL). We next compared the difference in EL- and LL-phase progesterone levels between the PMDD and control groups using Mann–Whitney U analysis in both the high- and low-estrogen groups. The results demonstrated that women with PMDD had a significantly higher EL-phase progesterone level in the low-estrogen group (Table 2 and Figure 1C), but not in the high-estrogen group. This result supported the moderating role of EL-phase estrogen level in the association between EL-phase progesterone level and PMDD.

## 4. Discussion

### 4.1. Lower EL-phase Estrogen Level in Women with PMDD

Women with PMDD had a lower EL-phase estrogen level than the controls in this presenting study. Studies have demonstrated no significant difference in ovarian hormone levels in women with PMDD [23,34]. However, a low sample size, evaluation during different menstrual phases, or a lack of control of the daily timing of blood drawing in the previous studies may explain this discrepancy. Thys-Jacobs et al. [24] demonstrated that women with PMDD had lower free estradiol level during the luteal phase. In the present study, the difference in LL-phase estrogen level just reached significance, but the difference in EL-phase estrogen level was robust. The rapid drop observed in the LL-phase level may have contributed to the large variation in estrogen levels. This would have attenuated the statistical power to determine the difference.

A previous review demonstrated a higher risk of mood symptoms among women whose estrogen levels are in a decreasing phase, such as the premenstrual phase, postpartum, and post-menopause [35]. Furthermore, tamoxifen, a medication that suppresses the estrogen function, contributes to depression [36]. These results suggest that withdrawal from estrogen contributes to mood symptoms and explains the lower LL-phase estrogen level among women with PMDD [24]. However, the lower EL-phase estrogen level of PMDD women in this study could limited the extent of the decline in estrogen in LL-phase and did not support the role of dynamic immediate estrogen withdrawal in PMDD symptom development. Further, PMDD symptoms are relieved after menstruation (Figure 1), which is the stage with the lowest estrogen level. If the estrogen withdrawal contributes to onset of PMDD symptoms. These symptoms would also not improve after onset of menstruation and may onset earlier than the LL phase.

Conversely, estrogen could modulate the function of a variety of neurotransmitters, such as serotonin and GABA [37], and inflammatory reaction [38], which determine vulnerability to depression and anxiety. Persistent low estrogen level in the luteal phase may contribute to the vulnerability of the women to emotional symptoms, but not directly to PMDD symptoms. For example, the lower estrogen in the luteal phase might contribute to susceptibility to inflammatory reaction and indirectly support the role of inflammation mechanism in PMDD [25]. However, these claims need to be evaluated in future studies.

### 4.2. Progesterone Level of Women with PMDD 

Progesterone has been repeatedly reported to exaggerate premenstrual symptoms in women with PMDD whose ovarian function has been suppressed [9,39]. Several studies have also tested the effect of add-back estradiol and progesterone among women with PMDD who were administered leuprolide, an agonist analogue of GnRH that suppresses ovarian function. Schmidt et al. [6] demonstrated that progesterone provokes premenstrual symptoms in women with PMDD but not in controls. Segebladh et al. [10] discovered that estrogen combined with progesterone provoked more premenstrual symptoms than estrogen only. These results suggest that PMDD symptoms are caused not only by progesterone fluctuation, but also the serum concentration and/or dose of estradiol and estradiol/progesterone ratio [10].

### 4.3. Interaction between EL-phase Estrogen and Progesterone Levels in PMDD Severity among Women with PMDD

In this study, covariant analysis demonstrated an interactive effect between EL-phase estrogen and progesterone levels on their association with PMDD severity. After controlling for the interaction term, EL-phase progesterone level associated with PMDD severity. This indicated that EL-phase progesterone level predicts LL-phase PMDD symptom severity. This might suggest a possible delayed effect of progesterone or a retention effect of progesterone level in the luteal phase. Further analysis indicated that the women with PMDD had higher EL-phase progesterone than the controls among the women with lower EL-phase estrogen levels, but not among those with higher EL-phase estrogen levels. These findings indicate that progesterone provoked PMDD symptoms, particularly among the women with lower EL-phase estrogen in the PMDD group.

One study demonstrated increased sensitivity to the GABA receptor agonist effect of allopregnanolone when administered to women with PMDD in the luteal phase [40]. This may explain why progesterone improves premenstrual symptoms in controls but provokes PMDD symptoms among women with PMDD. Our results suggested that a lower EL-phase estrogen level may be involved in the vulnerability to the progesterone effect in women with PMDD. Thus, combining the moderating effect of lower EL-phase estrogen level and the provoking effect of progesterone, the participants with PMDD experienced exacerbation of symptoms after a timely increase in progesterone level under a lower estrogen level. Subsequently, symptoms rapidly improved as the progesterone reached the lowest level after onset of menstruation.

Several limitations of this study should be noted. First, the number of participants was limited because numerous candidates were excluded due to the stringent criteria used for defining symptomatic cycles [32]. Second, there was variation in the duration of individual menstrual cycles, although there was no difference in the timing of blood tests during the evaluation cycle. Further, the EL and LL phase were estimated based on last menstruation period, not with ovulation test/LH levels. Finally, we asked the participants to rate their PMDD symptoms weekly, not daily, in the menstrual cycle. The effects of memory bias and mental averaging could thus not be prevented.

## 5. Conclusions

In summary, the women with PMDD exhibited lower EL-phase estrogen levels than the controls. We found that EL-phase estrogen level had an interactive effect on the association between EL-phase progesterone level and PMDD severity. Among the women with lower EL-phase estrogen levels, higher EL-phase progesterone was observed among the women with PMDD versus the controls. These results support that a low estrogen level contributes to vulnerability to the provoking effect of progesterone in the luteal phase among women with PMDD only. These results provide insight and may assist in the development of hormone intervention for treating PMDD. Further mechanistic studies are needed to understand the moderating role of low estrogen level in provoking PMDD symptoms among women with PMDD.

## Figures and Tables

**Table 1:** Age, educational level, phase of evaluation, and PMDD symptom severity in women with premenstrual dysphoric disorder (PMDD) and in controls.

Variables	PMDD Group (N = 63) (Mean ± SD)	Control Group (N = 53) (Mean ± SD)	*t*
Age (year)	25.02 ± 3.51	24.98 ± 3.73	0.052
Education level (year)	16.38 ± 1.41	16.11 ± 1.15	1.106
Early-luteal evaluation phase ^a^	0.64 ± 0.07	0.63 ± 0.10	−0.942
Late-luteal evaluation phase ^a^	0.92 ± 0.08	0.90 ± 0.08	−1.476
PMDD severity ^b^	68.83 ± 22.41	18.11 ± 21.27	12.426 ***

***: *p* < 0.001. ^a^ The evaluation date/duration of the menstrual cycle: the duration is calculated based on the date of menstruation before and after the evaluation cycle. ^b^ The score obtained from the premenstrual dysphoric disorder (PMDD) severity questionnaire. SD: standard deviation.

**Table 2:** Estrogen, progesterone (PG), luteinizing hormone (LH), and follicle-stimulating hormone (FSH) levels in the early-luteal (EL) and late-luteal (LL) phases among the PMDD and control groups.

Variables	PMDD Group (N = 63) (Mean ± SD)	Control Group (N = 53) (Mean ± SD)	Z ^a^
EL estrogen (pg/mL)	172.29 ± 89.76	241.50 ± 107.96	−3.71 ***
LL estrogen (pg/mL)	159.84 ± 102.55	202.72 ± 121.20	−2.05 *
EL PG (ng/mL)	9.63 ± 7.58	9.02 ± 7.69	−0.36
LL PG (ng/mL)	6.42 ± 6.40	7.93 ± 7.04	−1.47
EL LH (mIU/mL)	5.93 ± 4.85	5.34 ± 4.00	−0.42
LL LH (mIU/mL)	3.65 ± 2.46	3.96 ± 2.98	−0.67
EL FSH (mIU/mL)	3.35 ± 1.02	3.44 ± 1.21	−0.13
LL FSH (mIU/mL)	3.43 ± 1.93	3.36 ± 1.91	−0.25
**Low estrogen group**			
	**PMDD Group (N = 46)** **(Mean ± SD)**	**Control Group (N = 20)** **(Mean ± SD)**	
EL PG (ng/mL)	7.91 ± 6.37	4.08 ± 3.65	−2.11 *
LL PG (ng/mL)	7.14 ± 6.64	10.91 ± 7.59	−1.90
**High estrogen group**			
	**PMDD Group (N = 17)** **(Mean ± SD)**	**Control Group (N = 33)** **(Mean ± SD)**	
EL PG (ng/mL)	14.26 ± 8.77	12.02 ± 7.97	−1.03
LL PG (ng/mL)	4.50 ± 5.40	6.12 ± 6.12	−1.11

*: *p* < 0.05; ***: *p* < 0.001. ^a^ Z; Mann–Whitney test. PMDD: premenstrual dysphoric disorder. SD: standard deviation.

**Table 3:** The Spearman’s analysis for the correlation between estrogen, and progesterone in luteal phase among women with premenstrual dysphoric disorder (PMDD) and controls.

Pearson	EL PG	LLPG
PMDD group		
EL estrogen	0.45 ***	
LL estrogen		0.79 ***
Control group		
EL estrogen	0.56 **	
LL estrogen		0.84 **

**: *p* < 0.01; ***: *p* < 0.001; EL: early luteal; LL: late luteal; PG: progesterone. PMDD: premenstrual dysphoric disorder.

**Table 4:** General linear model univariable analysis of the effect of EL-phase estrogen level and EL-phase progesterone level on PMDD symptom severity ^a^.

	df	Mean Square	F test	η^2^
Model 1 (among all subjects)				
EL Estrogen	1	7668.54	7.163 **	0.060
EL Progesterone	1	2115.83	1.976	0.017
Model 2 (among all subjects)				
EL Estrogen	1	149.25	0.146	0.001
EL Progesterone	1	8486.97	8.295 **	0.069
EL estrogen X progesterone	1	6377.64	6.233 *	0.053
Model 3 (In PMDD group)				
EL Estrogen	1	488.74	1.019	0.017
EL Progesterone	1	2489.27	5.210 *	0.081
EL estrogen X progesterone	1	2700.03	5.631 *	0.087

*: *p* < 0.05; **: *p* < 0.01; df: degrees of freedom; η^2^: Eta squared; EL: early luteal; ^a^ The score obtained from the premenstrual dysphoric disorder (PMDD) severity questionnaire.
